# Combined transcript, proteome, and metabolite analysis of transgenic maize seeds engineered for enhanced carotenoid synthesis reveals pleotropic effects in core metabolism

**DOI:** 10.1093/jxb/erv120

**Published:** 2015-03-20

**Authors:** Mathilde Decourcelle, Laura Perez-Fons, Sylvain Baulande, Sabine Steiger, Linhdavanh Couvelard, Sonia Hem, Changfu Zhu, Teresa Capell, Paul Christou, Paul Fraser, Gerhard Sandmann

**Affiliations:** ^1^Unité de Biochimie et Physiologie Moléculaire des Plantes, INRA, 34060 Montpellier, France; ^2^School of Biological Sciences, Royal Holloway, University of London, Egham, Surrey TW20 OEX, UK; ^3^PartnerChip, Genopole Campus 2, 91000 Evry, France; ^4^Biosynthesis Group, Institute of Molecular Biosciences, Goethe University Frankfurt/M, Max von Laue Str. 9, D-60438 Frankfurt, Germany; ^5^Department of Plant Production and Forestry Science, University of Lleida-Agrotecnio Center, 25198 Lleida, Spain; ^6^Institució Catalana de Recerca i Estudis Avancats, 08010 Barcelona, Spain

**Keywords:** Genetically engineered carotenoid biosynthesis, GM maize, metabolomics, pathway regulation, proteomics, transcriptomics.

## Abstract

The aim of this study was to assess whether endosperm-specific carotenoid biosynthesis influenced core metabolic processes in maize embryo and endosperm and how global seed metabolism adapted to this expanded biosynthetic capacity. Although enhancement of carotenoid biosynthesis was targeted to the endosperm of maize kernels, a concurrent up-regulation of sterol and fatty acid biosynthesis in the embryo was measured. Targeted terpenoid analysis, and non-targeted metabolomic, proteomic, and transcriptomic profiling revealed changes especially in carbohydrate metabolism in the transgenic line. In-depth analysis of the data, including changes of metabolite pools and increased enzyme and transcript concentrations, gave a first insight into the metabolic variation precipitated by the higher up-stream metabolite demand by the extended biosynthesis capacities for terpenoids and fatty acids. An integrative model is put forward to explain the metabolic regulation for the increased provision of terpenoid and fatty acid precursors, particularly glyceraldehyde 3-phosphate and pyruvate or acetyl-CoA from imported fructose and glucose. The model was supported by higher activities of fructokinase, glucose 6-phosphate isomerase, and fructose 1,6-*bis*phosphate aldolase indicating a higher flux through the glycolytic pathway. Although pyruvate and acetyl-CoA utilization was higher in the engineered line, pyruvate kinase activity was lower. A sufficient provision of both metabolites may be supported by a by-pass in a reaction sequence involving phosphoenolpyruvate carboxylase, malate dehydrogenase, and malic enzyme.

## Highlight

Metabolomic, proteomic, and transcriptomic analysis of a maize line genetically engineered for enhanced seed carotenoid biosynthesis revealed how the sugar metabolism adapted to meet the additional precursor supply.

## Introduction

Carotenoids are essential nutritional factors of the human diet (Rao and Rao, 2007) which are needed for good vision in particular. Retinal, which is the light sensor in our eye, is derived by cleavage of carotenoids like β-carotene (as provitamin A) with an unsubstituted β-ionone end group. In addition, the yellow eye spot, the macula, protects the retina from UV-mediated oxidation. It consists of lutein and zeaxanthin. Only a constant supply of both nutritional carotenoids prevents the degradation of the macula which results in a loss of vision especially in elderly people (SanGiovanni and Neuringer, 2012). Several carotenoids are also important as food additives. The highest demands on natural and synthetic carotenoids relates to salmon and poultry farming (Tyczkowski and Hamilton, 1986).

Natural carotenoids originate from microbial or plant sources. Considerable attempts have been made to improve crop plants genetically for an increased accumulation of essential carotenoids. This includes cereals like rice (Ye *et al.*, 2000), potato (Römer *et al.*, 2002), fruit such as tomato (Fraser *et al.*, 2009) or oil-rich seeds such as canola (Shewmaker *et al.*, 1999). The carotenoids of interest were α- and β-carotene, lutein, and zeaxanthin. Recently, a library of maize transformants was constructed by the transfer of carotenogenic genes under endosperm-specific promoters for increased carotenoid production in the seeds (Zhu *et al.*, 2008). Among them was the maize line Ph3 that exhibited the highest carotenoid biosynthetic activity with a more than 100-fold higher content than the parent line.

Genetic engineering of a pathway for the higher accumulation of an end-product may have a global effect on the whole metabolism (Sandmann, 2001). Increased precursor utilization can negatively affect closely related pathways competing for the same precursors. One striking example is the dwarfisms of transgenic tomato plants with higher carotenoid synthesis in which geranylgeranyl pyrophosphate is diverted away from gibberellin biosynthesis (Fray *et al.*, 1995). In non-photosynthetic maize tissue, other pathways which, like carotenoid biosynthesis, rely on and compete for prenyl pyrophosphate utilization are γ-tocopherol and sterol biosynthesis. Although the latter class of compounds is synthesized via the mevalonate and not the deoxyxylulose 5-phosphate synthase (DXS) pathway, cross-talk of both pathways via prenyl pyrophosphate exchange is well established (Eisenreich *et al.*, 2001) and competition for storage space in the same membranes may have a regulatory effect on their biosynthesis. In general, decreased levels of certain metabolites from the early pathway may relieve regulatory constraints, readjusting the metabolite flow. In the case of enhanced carotenoid biosynthesis, changes in metabolism may occur in order to compensate for high influx into the plastidic DXS pathway leading to carotenoids. Transcriptomic, proteomic, and metabolomics/metabolite profiling are highly useful for detecting metabolic changes in transgenic plants (Ricroch *et al.*, 2011). A metabolomic approach revealed that increased carotenoid synthesis had a strong effect on global metabolite pools with an influence on fruit development in tomato (Fraser *et al.*, 2007). A deeper insight into the metabolic state of an organism can be obtained by parallel metabolomic and transcriptomic analyses (Enfissi *et al.*, 2010) or a combination of both techniques including proteomics (Barros *et al.*, 2010) leading to an understanding of how primary metabolism, in particular as a precursor source, is regulated and adapted to enhanced carotenoid bioynthesis. This is an important issue for the biotechnological production of carotenoids in genetically engineered plant tissues. Therefore, in the present investigation, direct analysis of relevant terpenoids, metabolomics, transcriptomics, and proteomics was applied. This integrative approach revealed the metabolic changes in transgenic Ph3 maize with enhanced carotenoid biosynthesis compared with the wild-type line. Our results identified the key adaptations of sugar metabolism which are necessary to fuel the higher through-put via the carotenoid pathway.

## Materials and methods

### Maize material

In previous work, three independent transformant lines were selected by demonstrating combined *psy*1 and *crtI* gene integration, accumulation of their transcripts, and very similar enhanced carotenoid synthesis (Zhu *et al.*, 2008). The Ph3 line with the highest total carotenoid content used here exhibited normal growth and unaffected germination behaviour compared with the wild type and also to the other two lines with the same transgene expressions. All maize plants were grown in the greenhouse with a 10h photoperiod (28/20 °C day/night temperature) and 60–90% relative humidity for 50 d, followed by a 16h photoperiod (21/18 °C day/night temperature) thereafter, and self-pollinated. Endosperm and embryo were separated and collected at 30 d after pollination (DAP), frozen in liquid nitrogen, and stored at –80 °C until use.

### Metabolite analysis: carotenoid and terpenoid analysis

The powdered seeds, endosperm, and embryo were extracted with tetrahydrofuran/methanol (50:50, v/v) by heating for 20min at 60 °C. After partitioning into 30% ether in petrol, the upper phase was collected, evaporated, and re-dissolved in acetone methanol for high-performance liquid chromatography (HPLC) analysis. A similar HPLC approach for simultaneous determination of carotenoids, sterols, and tocopherols described by Schüep et al. (1995) was used. This was achieved on a 15cm Nucleosil C18, 3μ column with a mobile phase of acetonitrile/2-propanol/methanol/water (82:5:10:3, by vol.) and a flow rate of 1ml min^–1^ at 20 °C column temperature. Spectra were recorded online with a photodiode array detector 440 (Kontron, Straubenhard, Germany). Detection wavelengths were 205nm for sterols, 295nm for γ-tocopherol, and 450nm for carotenoids. Identification and quantification was performed by co-chromatography and comparison of spectral properties with authentic standards and reference spectra (Britton *et al.*, 2004).

### Metabolome analysis

General metabolite profiling of polar and non-polar metabolites was performed in accordance with the procedures described in Enfissi *et al.* (2010). In brief, methanol (1ml; HPLC grade) and the internal standard ribitol (20mg ml^–1^) were added to freeze-dried (10–25mg) of maize tissue powder. The suspended material was mixed vigorously and then incubated at room temperature for 30min with continuous agitation. To remove cell debris, samples were centrifuged at 12 000rpm for 2min. The resulting supernatant was removed and, from each sample, an aliquot (100 μl) removed and dried completely under nitrogen gas. At this point, the extracts could be derivatized immediately or stored without degradation at −20 °C. Derivatization was performed by the addition of 30 μl methoxyamine–HCl (Sigma-Aldrich) prepared at a concentration of 20mg ml^–1^ in pyridine. After incubation in screw-capped tubes at 37 °C for 2h, *N*-methyl trimethylsily-trifluoro-acetamide from Macherey Nagel was added (85 μl) and the samples incubated for a further 1h at 37 °C before analysis. Gas chromatography–mass spectrometry analysis was performed on an Agilent HP6890 gas chromatograph with a 5973MSD. Typically, samples (1ml) were injected with a split/splitless injector at 290 °C with a 20:1 split and repeated on a 200:1 split for sugar quantification. Retention time locking to the internal standard was used. The gas chromatography oven was held for 4min at 70 °C before ramping at 58 °C min^–1^ to 310 °C. This final temperature was held for a further 10min, making a total time of 60min. The interface with the MS was set at 290 °C and MS performed in full scan mode using 70eV EI+ and scanned from 10–800 D. To identify chromatogram components found in the maize profiles, a mass spectral (MS) library was constructed from in-house standards as well as the NIST 98MS library. Chromatogram components were initially processed by the automated MS de-convolution and identification system. A retention time calibration was performed on all standards to facilitate the determination of retention indices (RIs). Using the retention indices and MS, identification was performed by comparison with the MS library. Quantification was achieved using Chemstation (Agilent) software facilitating integrated peak areas for specific compound targets (qualifier ions) relative to the ribitol internal standard peak.

### Transcriptome analysis

Biological replication for transcriptome analysis was created using a minimum of five plants. Total RNA was isolated from 30 DAP maize endosperm using the RNeasy Plant Mini Kit (QIAGEN, Valencia, CA, USA) and residual DNA was removed with DNase I. The quality of the RNA was assessed by capillary electrophoresis (RNA 6000 Nano chips, Agilent) and used for the synthesis of cDNA which was transcribed *in vitro* to produce multiple copies of biotin-modified amplified RNA according to the protocols of the manufacturer (3′ IVT Express kit, Affymetrix). After a purification step and RNA fragmentation, hybridization on GeneChip 3′ expression arrays was carried out overnight. Staining using streptavidin-phycoerythrin conjugate and subsequent washing steps were performed using the Affymetrix fluidic station 450 and arrays were finally scanned with the Affymetrix GeneChip Scanner 3000. Probe-level expression data (CEL files) were finally produced using GeneChip Command Console software. Affymetrix quality controls of raw data intensities were assessed using GeneChip Expression Console software. Statistical analysis was then carried out using GeneSpring GX12 software. First, quantitative normalization was performed using the GC-RMA algorithm and then differentially expressed genes were identified using a modified *t* test implemented in the software based on statistical significance (*P* value <0.05) and fold-change (FC >2). Transcriptome and also proteome data were visualized using Volcano plots (Li et al., 2012).

## Proteome analysis

Ground maize endosperms were suspended in extraction buffer (100mM TRIS-HCl pH 6.8, 100mM DTT, 20% glycerol, 5% SDS, 5mM EDTA, and 1% protease inhibitor SIGMA P9599) by stirring for 40min followed by sonication. After centrifugation, proteins were precipitated from the supernatant with ice-cold acetone (1:4 v/v, –20 °C) and centrifuged again. The protein pellets were washed and re-suspended in 500 μl of Laemmli buffer 1× (0.1M TRIS-HCl pH 6.8, 0.1M DTT, 10% glycerol, 2% SDS) and their concentrations calculated with the 2-D Quant Kit (Amersham Biosciences, GE Healthcare).

Proteins (15 μg) were fractionated by one dimensional SDS-polyacrylamide gel electrophoresis (PAGE) on 12% gels. In-gel protein digestion was carried out overnight at 37 °C with trypsin (Trypsin Gold, Promega) at an enzyme to protein ratio of 1:60 in bicarbonate buffer,. Peptides were extracted by incubating the bands in 80% acetonitrile (ACN) and sonication. Solutions containing peptides were evaporated and peptides solubilized in 10 μl of 2% formic acid. In label-free experiments, all samples were processed for the same time and on the same gel ensuring the same conditions. This allows relative quantification even if the trypsin digestion was incomplete.

Total protein (250ng) of each sample was analysed by mass spectrometry using a Q-TOF mass spectrometer (Maxis Impact, Bruker Daltonics) with a Captive Spray ion source interfaced with a nanoLC Ultimate 3000 (Thermo Scientific). Samples were concentrated on a pre-column (C18, 300 μm×5mm, Thermo Scientific) at a flow rate of 20 μl min^–1^ using 0.1% formic acid and then separated with a reversed-phase capillary column (C 18 PepMan10, 75 μm×250mm, 3 μm, 100A, Thermo Scientific) at a flow rate of 0.3 μl min^–1^ using a two steps gradient (8% to 28 % ACN with 0.1% formic acid in 40min then 28% to 42% in 10min), and eluted directly into the mass spectrometer.

Proteins were identified by MS/MS by information-dependent acquisition of fragmentation spectra of multiple charged peptides using Data Analysis software (Bruker Daltonic GmbH, Bremen, Germany) to generate peak lists. Protein identification was obtained by searching with a Mascot server (v.2.2.07, Matrix Science) against theSequenceMaize.org and UniProtKB (maize taxonomy) combined database, including as a fixed modification: carbamidomethylation of cysteine, and as variable modifications: N-terminus acetylation, deamidation of asparagine and glutamine, oxidation of methionine, and phosphorylation of serine, threonine, and tyrosine.

Label-free quantification was done with Ideal-Q software (Tsou *et al.*, 2010). This tool normalizes the area under the curve from the extracted ion chromatogram by dividing the area of each quantified peptide by the sum of the areas of all quantified peptides within its LC-MS run. Since a peptide can be detected in several SDS-PAGE bands, peptide abundance in one sample is calculated by summing the normalized areas of this peptide in each of these bands (Gautier *et al.*, 2012).The peptide abundance in one biological condition was determined by the average of its area values in the different biological replicates. At the protein level, unmodified specific peptides present in at least three of the four biological replicates were used to calculate a protein quantitative ratio. Protein abundance in each experimental condition was calculated by summing peptide abundances. The ratio of the protein and the corresponding Student *t* test between the different experimental conditions were calculated.

## Results

**Fig. 1.**
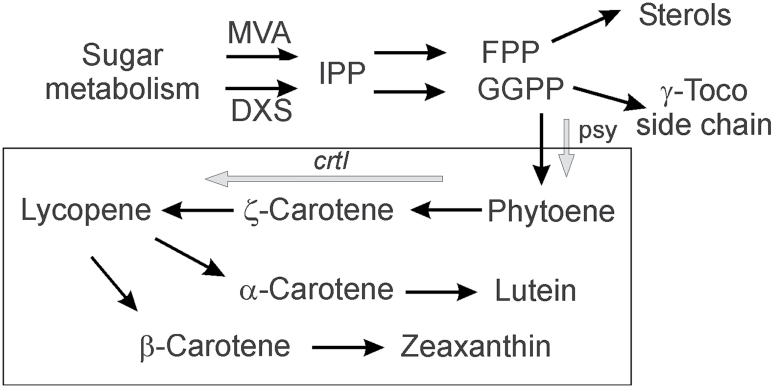
Endosperm-targeted genetic engineering of carotenoid biosynthesis in maize kernels. The specific carotenoid pathway is boxed, grey arrows indicate the transformation steps.

Genetic engineering of the carotenoid pathway was carried out by transformation with additional copies of the endogenous phytoene synthase gene *psy*1 from maize in combination with a bacterial desaturase gene (*crtI*) (Zhu *et al.*, 2008) which performs the entire desaturation series in the pathway (Fig. 1). Both genes were expressed under different endosperm-specific promoters, the wheat low-molecular-weight glutenin (Colot *et al.*, 1987) and the barley D-hordein promoter (Sørensen *et al.*, 1996). In addition, the *bar* gene encoding phosphinothricin *N*-acetyltransferase (Thomson *et al.*, 1987) was used as a selectable marker.

### Targeted terpenoid analysis and metabolite profiling

Since the genetic manipulation was endosperm targeted, carotenoid composition and the concentrations of other terpenoids, sterols, and tocopherol were analysed in the endosperm and embryo (Table 1). More than 90% of the carotenoids in the non-transgenic variety M37W are located in the embryo. The dominating carotenoids are zeaxanthin and its diepoxide violaxanthin. There, the accumulation of α-carotene and its hydroxy derivative lutein was below detection. The biosynthesis engineered into the endosperm of the transgenic line Ph3 enhanced carotenoid biosynthesis by more than 20-fold in this part of the seed. The carotenoids with the highest concentrations were zeaxanthin and its monohydroxylated direct precursor β-cryptoxanthin. This increase of carotenoid synthesis in the endosperm works partially at the expense of the synthesis in the embryo which was only half of that found in the non-transgenic line. Another change in the embryo carotenoid composition was the occurrence of lutein and α-cryptoxanthin in Ph3. When seeds reached 60 DAP, carotenoids had accumulated further, especially in the endosperm, by up to 20%. This increase was mainly due to zeaxanthin and β-carotene (data not shown).

**Table 1.** Terpenoids (μg g^–1^ dw) in seed embryo and endosperm of maize wild type M37W and transformant Ph3 at 30 d after pollination

	Wild type M37W		Ph3	
	Endo^*b*^	Emb^*b*^	Endo	Emb
Carotenoids				
Neoxanthin	0.22±0.01^*a*^	2.17±0.46	1.96±0.15	0.85±0.22
Violaxanthin	0.83±0.05	8.69±2.47	7.72+0.58	2.89±0.10
Lutein	0.41±0.03	nd	3.92±0.30	1.38±0.03
Zeaxanthin	1.18±0.08	18.68±4.23	27.46±2.05	5.61±0.15
α-Cryptox^*b*^	0.06±0.01	nd	6.28±0.48	0.75±0.02
β-Cryptox^*b*^	0.03±0.01	2.58+0.71	11.48±0.99	1.12±0.03
β-Carotene	0.16±0.01	1.12+0.29	3.70±0.29	1.99±0.05
Total	2.89	33.32	62.52	14.59
Sterols				
Stigmasterol	173±37	804±208	216±40	706±168
Sitosterol	297+41	624±176	330±51	1408±212
Total	470	1428	546	1814
*γ-Toco* ^*c*^	nd	61.20+7.51	nd	44.32+8.10

^*a*^ Mean ± SD from five samples.

^*b*^ Abbreviations: Endo, endosperm; emb, embryo; α-Crypotox, α-cryptoxanthin; β-Cryptox, β-cryptoxanthin.

^*c*^ May include trace amounts of β-tocopherol.

Other terpenoid or terpenoid-related pathways were also affected by carotenogenesis engineering, particularly in the non-targeted embryo (Table 1). Tocopherols are derived from two pathways, the shikimate pathway for the synthesis of the chromanol ring and the terpenoid pathway which provides the hydrocarbon side-chain (Mène-Saffrané and DellaPenna, 2010). γ-Tocopherol was found exclusively in the embryo and not in the endosperm. It was the only tocopherol detectable in seeds harvested 30 DAP. However, in seed embryos at 60 DAP, traces of α-tocopherol were found and the total tocopherol content was about 10% higher.

Sterols in maize kernels are present predominantly as free sterols or as sterol fatty acid esters (Davis and Poneleit, 1974). Since saponification was used during extraction, our data indicate the sum of free and esterified sterols. The major sterols identified were stigmasterol and sitosterol (Table 1). They accumulated in the endosperm and in higher concentrations in the embryo. The only observed difference between M37W and the transgenic line was an increase of the sitosterol content in the embryo of Ph3 which is responsible for higher total sterols therein. In older seeds (60 DAP), the sterol content did not change significantly.

**Fig. 2.**
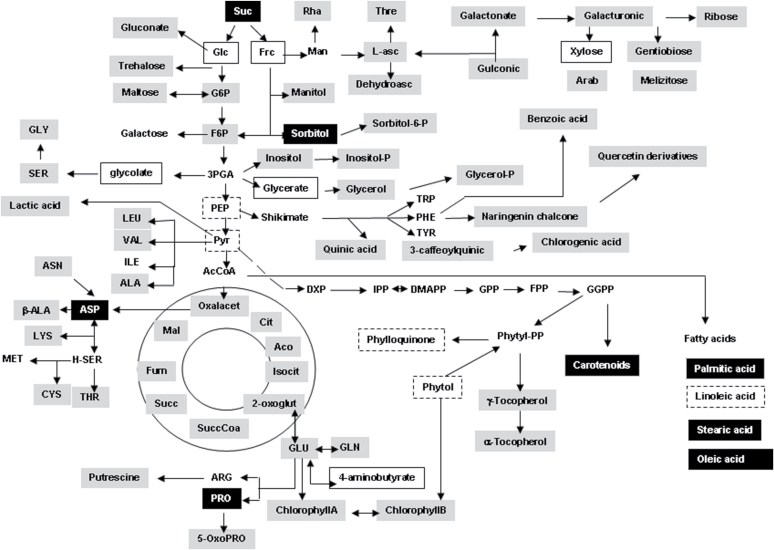
Pathway map visualizing metabolite changes in maize seeds resulting from the genetic engineering of carotenoid biosynthesis. The pathway diagram was created using the in-house software BioSynlab. Metabolites on a grey background or otherwise marked were detectable with this platform, compounds in dotted boxes were below detection. Compounds on a black background indicate an at least 2-fold significant increase in the transgenic line Ph3 over the non-transgenic line M37W. The boxed compounds on a white background exhibited an at least 2-fold significant decrease.

To investigate global changes of metabolites after genetic modification of carotenogenesis, metabolite profiling of the intermediary metabolism was carried out. The platform used for metabolomic analysis is described in Enfissi *et al.* (2010) and Perez-Fons *et al.* (2014) and detects most of the compounds shown in Fig. 2 where they are marked accordingly. This figure depicts the results of the non-targeted metabolite analysis of kernels showing changes of individual compounds by more than 2-fold in transgenic Ph3 compared with the wild type, M37W. Among the sugars, sucrose and sorbitol had increased and glucose, fructose, and xylose had decreased in Ph3. Other metabolites from early glycolysis with changed concentrations were glycolate and glycerate which were lower in Ph3. In addition, the two amino acids aspartate and proline had increased in Ph3. Three fatty acids were detectable—palmitic, stearic, and oleic acids—with a greater than an 8-fold increase. Additional information of changing metabolite pools can be found in Supplementary Table S1 at *JXB* online.

### Proteomic and transcriptomic profiling

**Fig. 3.**
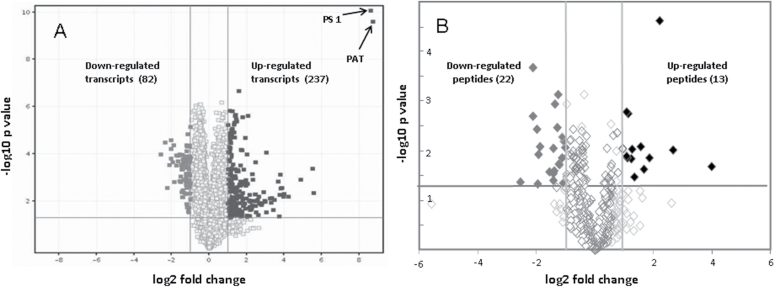
Volcano plots of up- and down-regulated transcripts (A) and peptides (B) in the endosperm of Phe3 with genetically engineered carotenoid biosynthesis compared with wild-type M37W.

Proteomic profiling of endosperm from M37W and transgenic Ph3 included protein fractionation, tryptic digestion, peptide separation, and analysis by tandem mass spectrometry. The fragmentation spectra were used for the identification of the corresponding proteins in the database. Proteins were validated once they were identified with two peptides having a Mascot score *P* value <0.05. Our approach allowed the identification of 1394 non-redundant proteins in M37W and 1350 in transgenic Ph3. For data quantification (label-free), Ideal-Q software (Tsou *et al.*, 2010) combined with an in-house programme was used. In total, 24 proteins varied in their abundance corresponding to the quantified peptides (35 peptides) (Fig. 3A). Proteins with a significant quantitative change (*P* <0.05) and with a fold-change of 2 were considered. Ten of them were significantly increased and 14 decreased. The relevant enzymes of carbohydrate metabolism, providing the precursor for carotenoid biosynthesis, which increased in transgenic Ph3, were glucose-6-phosphate isomerase, fructose-*bis*phosphate aldolase, and phosphoenolpyruvate carboxylase (Table 2A). By contrast, the amounts of sucrose synthase, pyruvate kinase, UDP-glucosyltransferase and pyruvate phosphate dikinase were decreased. Other enzymes which were increased or decreased can be found in Supplementary Table S2 at *JXB* online.

**Table 2.** Changing proteins and transcripts related to the sugar metabolism in the maize kernel endosperm 30 d after pollination of Ph3 versus wild type

Accession	Enzyme, EC number	Ratio Ph3/WT
Protein^*a*^		
sp|P49105|G6PI_MAIZE	Glucose 6-phosphate isomerase, cytosolic EC 5.3.1.9	3.13*
tr|B4FWP0|B4FWP0_MAIZE	Fructose *bis*phosphate aldolase EC 4.1.2.13	2.54*
tr|D2IQA1|D2IQA1_MAIZE	Sucrose synthase EC 2.4.1.13	0.47
tr|B4F9G8|B4F9G8_MAIZE	Pyruvate kinase EC 2.7.1.40	0.34**
tr|C0LNQ9|C0LNQ9_MAIZE	UDP-glucosyltransferase EC 2.4.1.35	0.27**
tr|Q9SAZ6|Q9SAZ6_MAIZE	Phosphoenolpyruvate carboxylase EC 4.1.1.31	2.12*
tr|K7UVD7|K7UVD7_MAIZE	Pyruvate phosphate dikinase EC 2.7.9.1	0.25**
Transcript	Selection marker	
	Phosphinotricine acetyltransferase	
Zm.19140.1.A1_at	Phytoene synthase EC 2.5.1.32	399.4**
Zm.2869.1.A1_at	Asparagine synthetase EC 6.3.5.4	3.0**
Zm.6169.1.A1_at	Fructokinase EC 2.7.1.4	2.4**
Zm.26.1.A1_at	Sucrose phosphate synthase EC 2.4.1.14	0.4**

^*a*^ By peptide identification and quantification.

^*b*^ Asterisks: *P <0.1; **P <0.05.

The same maize endosperm material was used to quantify transcript accumulation in M37W and Ph3. Gene expression was determined with an Affymetrix GeneChip Maize Genome Array. It contains 17 555 probe sets to interrogate approximately 14 850 maize transcripts which represent 13 339 genes covering about one-third of the maize genes (Schnable *et al.*, 2009). Differential expression was obtained for 238 up-regulated and 82 down-regulated transcripts comparing Ph3 versus M37W (Fig. 3B). One transcript specifically detected in Ph3 is that of the *bar* gene encoding the selection marker encoding phosphinothricin *N*-acetyltransferase (PAT). The highest quantitative transcripts increase was that of the *psy1* gene which was the over-expressed maize gene (Fig. 1). Four genes associated with carbohydrate metabolism were differentially expressed in M37W and Ph3 are listed in Table 2B. Transcript abundance for asparagine synthetase and fructokinase increased, whereas that of sucrose phosphate synthase decreased. Other transcripts differentially expressed in M37W and Ph3 are listed in Supplementary Table S3 at *JXB* online.

### Combination of data related to sugar metabolism

**Fig. 4.**
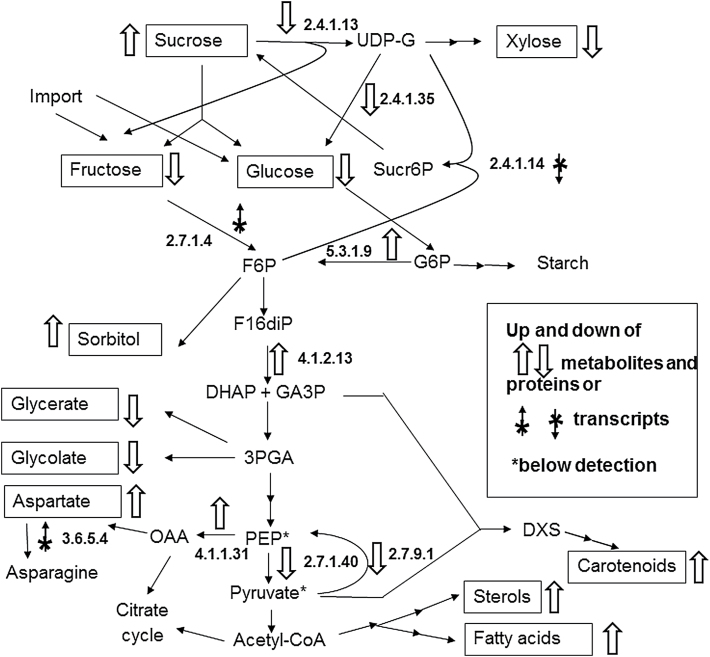
Concentration changes of metabolites, proteins, and transcripts in the sugar metabolism of maize kernels in the transgenic line Ph3 versus wild-type line M37W. Boxes indicate changed metabolite pools, open arrows indicate their up- or down-regulation. Arrows next to a reaction, in combination with the EC number, indicate changes in protein concentrations or levels of transcripts. An asterisk indicates metabolites below the detection level. Abbreviations: UDP-G, UDP-glucose; F6P, fructose 6-phosphate; G6P, glucose 6-phosphate; F16diP, fructose 1,6-*bis*phosphate; DHA, dihydroxyacetonphospate;GA3P, glyceraldehyde 3-phosphate; 3PGA, 3-phospoglycerate; OAA, oxaloacetate; PEP, phosphoenolpyruvate; DXS, deoxyxylulose 5-phosphate.

Precursors for the enhanced synthesis of carotenoids, sterols, and fatty acids are generated by sugar metabolism. This involves the import of sugars or the mobilization of storage products, glycolysis, and the provision of glyceraldehyde phosphate, pyruvate, and acetyl-CoA. Figure 4 shows the metabolism of sugar together with related amino acids and the changes observed in Ph3 by comparing the metabolite pools (Table 1; Fig. 2), transcript and protein concentrations (Fig. 3). Hexose phosphates, such as glucose 6-phosphate and fructose 6-phosphate, are key metabolites for glycolysis or the synthesis of storage reserves, such as sucrose or starch, in cereals. In transformant Ph3, the sucrose pool is higher than in M37W. This coincides with decreased activities of the enzymes participating in the metabolism of sucrose to xylose, glucose, and fructose. These three sugars showed a lower pool size in Ph3. This may be due to the decreased enzyme amounts found for sucrose synthase (EC 2.4.1.13) and UDP-glucosyltransferase (EC 2.4.1.35).

Maize kernels as a sink tissue are supported by assimilates from outside. Fructose and glucose are the main imported carbohydrates into the embryo and endosperm (Alonso et al., 2011). Both sugars were found in lower concentrations in Ph3. This may be due to a more efficient formation of fructose-6-phosphate from fructose indicated by the higher transcript level of fructokinase (EC 2.7.1.4). In addition, enhanced metabolism of glucose via glucose 6-phosphate to fructose 6-phosphate may be supported by enhanced activity of glucose 6-phosphate isomerase (EC 5.3.1.9). In addition, expression of the gene of sucrose 6-phosphate synthase (EC 2.4.1.14), responsible for the formation of sucrose 6-phospate from UDP-glucose, and fructose 6-phosphate were lower which limits the drain of fructose 6-phosphate towards the synthesis sucrose. Fructose-6-phosphate is directly converted to sorbitol which was found in higher concentrations in Ph3. The main utilization of fructose-6-phosphate, however, is as the substrate for glycolysis. Along the glycolytic pathway, one enzyme fructose 1,6-*bis*phosphate aldolase (EC 4.1.2.13) was present in higher concentrations in Ph3 which may indicate a higher metabolic flux to trioses including glyceraldehyde 3-phosphate, one of the precursors for carotenoid synthesis, and further on to phosphoenolpyruvate. Withdrawal of the glycolysis intermediate 3-phosphoglycerate for the formation of glycerate and glycolate may be reduced in Ph3 as judged from the lower pools of both compounds. Phosphoenolpyruvate is another central metabolite generated in the glycolytic pathway. In Ph3, it feeds into oxaloacetate through higher concentrations of phosphoenolpyruvate carboxylase (EC 4.1.1.31) and increases the aspartate pool. Also the higher transcript levels of asparagine synthetase (EC 6.3.5.4) indicate stronger synthesis of asparagine. For the synthesis of carotenoids or sterols and fatty acids, phosphoenolpyruvate must be converted to pyruvate. However, decreased enzyme amounts for pyruvate kinase (EC 2.7.1.40) do not fit with a higher pyruvate and acetyl-CoA demand in Ph3. However, pyruvate phosphate dikinase (EC 2.7.9.1), using pyruvate in the reverse reaction to phosphoenolpyruvate, is also present in lower concentrations.

## Discussion

### Terpenoid synthesis in endosperm and embryo of maize kernels

The crucial step of increasing carotenoid content in Ph3 was the over-production of phytoene synthase (Figs 1; 3A) which has been shown in different plant species to limit carotenoid synthesis (Sandman et al., 2006). Enhancement of carotenoid synthesis also affected by other unrelated pathways, in particular sterols and fatty acids synthesis. Their synthesis differs in maize embryo and endosperm, which are part of the kernels, to about 10% and 90%, respectively. The embryo is metabolically more active. γ-Tocopherol was found exclusively in the embryo which demonstrates a precise separation of both compartments (Table 1). The embryo is the major site of carotenoid biosynthesis. In this tissue, carotenoid concentration is more than 10-fold enriched compared with the endosperm and the sterol content is also 3-fold higher in the embryo when compared with the endosperm. This corresponds to the accumulation of *Psy1* transcripts in maize embryo (Singh *et al.*, 2003). The metabolomic data indicate a higher rate of fatty acid synthesis (Fig. 2) which should also occur in the embryo since it is the compartment of oil biosynthesis (Barthole *et al.*, 2012). In the transgenic line Ph3, carotenoid formation is engineered in the endosperm (Zhu *et al.*, 2008). This not only enhanced carotenoid biosynthesis in the endosperm making this tissue the major carotenogenic site in the kernel but, in addition, decreased carotenoid synthesis in the embryo (Table 1). Unexpectedly, sterol and fatty acid synthesis were also higher in the transgenic line. It should be pointed out that all three pathways are competitive to each other and operate in two different tissue compartments (Table 1).

### Adaptation of the sugar metabolism in the transformant Ph3 according to higher upstream utilization

Enhancing product formation in a biosynthesis pathway by genetic engineering has a pronounced impact on the entire metabolism. In general, plants have the flexibility to adapt their metabolic network to a higher metabolite provision for consumption by the extended pathway activities. In the present study, it has been shown that the transgenic line Ph3 needs a higher flux through and out of the glycolytic pathway for the synthesis of carotenoids, sterols, and fatty acids.

Metabolic flexibility to cope with an extended biosynthesis capacity is provided by co-ordinated changes of the metabolite fluxes. It can be assumed that the flux through the entire metabolite chain into the increased pathway end-products is determined by several enzymes. Different factors can influence the rate determining the contribution of an enzyme (Kacser and Porteous, 1987; Morandini, 2013). One factor is its enzyme concentration/activity which is effective only when the enzyme catalyses a first order reaction making it sensitive to changes in substrate concentration (indicated by flux control coefficients close to one). Another factor is sensitivity to changes in substrate concentrations (concentration control). This depends on its kinetic characteristics and can only be observed when the substrate concentration is far below the *K*_m_ value. To understand metabolic control and adaptation leading to the increased supply of the demand site in Ph3, we looked at the changes in metabolite pool sizes and the concentration of enzymes determined directly or via transcript levels as indicators. In the case of the enhanced pathways, glycolysis connects supply and demand by an accelerated flow into and through the glyceraldehyde 3-phosphate and phosphoenolpyruvate / pyruvate pools (Fig. 4). It should be pointed out that the present analysis did not show any indication for changes in the pentose phosphate pathway in the transformant Ph3.

From the integrated data, the following model scenario can be assumed. Carbon supply of maize kernels is provided by the import of fructose and glucose (Alonso et al., 2011). The pools of both sugars were depleted by higher activities of fructokinase (EC 2.7.1.4) as well as glucose 6-phosphate isomerase (EC 5.3.1.9) which initiates higher glycolytic flux through fructose 6-phosphate. Since starch synthesis competes with glycolysis for glucose 6-phosphate, enhanced activities of glucose 6-phosphate isomerase favour the flow into glycolysis. Another competitive reaction using glucose 6-phosphate is catalysed by sucrose 6-phosphate synthase (EC 2.4.1.14) which exhibited decreased activity in Ph3. In parallel, the sucrose metabolism to glucose and fructose seems to have a minor contribution to glycolytic activity in Ph3 due to lower activities of sucrose synthase (EC 2.4.1.13) and UDP-glucosyl transferase (EC 2.4.1.35). Decreased sucrose conversion and decreased synthesis of xylose may be the reason for a higher sucrose pool in the transgenic maize line.

In the central section of the glycolytic pathway, fructose 1,6-*bis*phosphate aldolase (EC 4.1.2.13) exhibited a higher activity in Ph3. This is an allosteric enzyme controlled by phosphoenolpyruvate in plants (Hodgson and Plaxton, 1998). Further downstream, the activity of phosphoenolpyruvate kinase (EC 2.7.1.40) is decreased in Ph3, although the resulting pyruvate from this reaction is an essential building block for the DXS pathway to carotenoids. In addition, acetyl-CoA, the final product of glycolysis, is needed for sterol and fatty acid synthesis (Fig. 4). Metabolism of phosphoenolpyruvate is highly complex making an interpretation of the fluxes extremely difficult. Looking at phosphoenolpyruvate kinase, this virtually irreversible enzyme is far from equilibrium (Nelson and Cox, 2008) and, therefore, a prominent candidate for flux control by regulation. Indeed, it is allosterically down-regulated particularly by intermediates of the citrate cycle (Knowles *et al.*, 2001; Plaxton *et al.*, 2002). As a supporting alternative in maize kernels, pyruvate formation can by-pass pyruvate kinase catalysis by a reaction sequence involving phosphoenolpyruvate carboxylase, malate dehydrogenase, and malic enzyme (Detarsio *et al.*, 2008). The first enzyme in this alternative route, phosphoenolpyruvate carboxylase (EC 4.1.1.31), is present in higher activities in Ph3. In addition, lower activities of pyruvate phosphate dikinase (EC 2.7.9.1) reduce the back reaction of pyruvate to phosphoenolpyruvate.

It is difficult to understand the metabolic up-regulation of acetyl-CoA-dependent sterol and fatty acid synthesis in connection with the engineered carotenoid biosynthesis pathway (Fig. 4). A possible explanation is that, in the wild type, both reaction branches are not saturated with acetyl-CoA. Therefore, synthesis is substrate-driven and a higher provision of acetyl-CoA results in increasing synthesis rates and enhanced formation of their upstream end-products. Furthermore, the mevalonate and DXS terpenoid pathways (to sterols and carotenoids, respectively) are interconnected via the translocation of prenyl pyrophosphates (Eisenreich *et al.*, 2001; Bick and Lange, 2003) which may also allow a simultaneous flux increase into both pathways.

### Implications on future engineering of the carotenoid pathway

Our integrated approach is a first step in understanding how maize kernels engineered for higher carotenoid biosynthesis change their metabolism in response to an increasing metabolite flow into this and other pathways. After the over-expression of *psy1* encoding phytoene synthase, the gateway enzyme for carotenogenesis (Sandman et al., 2006), was successful in increasing carotenoid synthesis, the next step for further enhancement should target deoxyxylulose 5-phosphate synthase (Estevez et al., 2001). However, this will increase the demand for precursors even further. Therefore, an attempt was made to find putative flux limitations in primary metabolism which feeds into terpenoid and fatty acid biosynthesis. Promising enzyme targets may be: (i) invertase increasing sucrose metabolism towards gylcolysis; (ii) the already up-regulated enzyme glucose 6-phosphate isomerase which provides metabolites for glycolysis but competes with starch synthesis for glucose 6-phosphate; and (iii) fructose 1,6-diphosphate aldolase for additionally higher glycolytic activity. Alternatively, over-expression of the pyruvate kinase gene may help to relieve a developing limitation of the pyruvate and acetyl-CoA supply for the synthesis of terpenoids and fatty acids. Over-expression of selected genes can lead not only to a deeper insight into the adaptation process caused by engineering of the carotenoid pathway but may also fortify the precursor supply needed for higher metabolite flow into genetically further enhanced carotenogenesis.

## Supplementary data

Supplementary data can be found at *JXB* online.

Supplementary Table S1. List of all up- and down-changing metabolites in maize line Ph3

Supplementary Table S2. List of significantly changed proteins in transgenic maize line Ph3.

Supplementary Table S3. List of the 10 highest up- and down-changing transcripts in transgenic maize line Ph3.

Supplementary Data
