# Supplementary material for: Combined transcript, proteome, and metabolite analysis of transgenic maize seeds engineered for enhanced carotenoid synthesis reveals pleotropic effects in core metabolism
Source: J Exp Bot. 2015 Mar 20;66(11):3141–50. doi: 10.1093/jxb/erv120 (PMC4449536; doi:10.1093/jxb/erv120)
Supplement: Supplementary Data [file supp_66_11_3141__index.html]

Combined transcript, proteome, and metabolite analysis of transgenic maize seeds engineered for enhanced carotenoid synthesis reveals pleotropic effects in core metabolism — Combined transcript, proteome, and metabolite analysis of transgenic maize seeds engineered for enhanced carotenoid synthesis reveals pleotropic effects in core metabolism — Supplementary Data 

# Combined transcript, proteome, and metabolite analysis of transgenic maize seeds engineered for enhanced carotenoid synthesis reveals pleotropic effects in core metabolism

## Supplementary Data

Data files

**Files in this Data Supplement:**

- Supplementary Data - Supplementary Data
