# Supplementary material for: Combined transcript, proteome, and metabolite analysis of transgenic maize seeds engineered for enhanced carotenoid synthesis reveals pleotropic effects in core metabolism
Source: J Exp Bot. 2015 Mar 20;66(11):3141–50. doi: 10.1093/jxb/erv120 (PMC4449536; doi:10.1093/jxb/erv120)
Supplement: Supplementary Data [file supp_erv120_jexbot141762_file001.pdf]

Combined transcript, proteome and metabolite analysis of transgenic maize seeds engineered for enhanced carotenoid synthesis reveals pleotropic effects in core metabolism . *Mathilde Decourcelle, Laura Perez-Fons, Sylvain Baulande, Sabine Steiger, Linhdavanh Couvelard, Sonia Hem, Changfu Zhu, Teresa Capell, Paul Christou, Paul Fraser, and Gerhard Sandmann*

SUPPLEMENTARY DATA

Supplementary Table S1. *List of all up and down changing metabolites in maize line Ph3*

CAS number	Description	Ratio Ph3 / WT	P-value*
57-10-3	Palmitic acid	20.0	1.0E-04
112-80-1	Olelic acid	16.0	1.0E-04
57-11-4	Stearic acid	15.0	1.0E-04
50-70-4	Sorbitol	10.0	1.0E-04
2058-58-4	Asparagine	10.0	1.0E-04
50-70-4	Sucrose	3.0	1.0E-03
540-04-5	Phytoene	2.0	5.0E-02
7615-88-5	phytofluene	1.5	5.0E-02
60-33-3	Linoleic acid	1.2	5.0E-02
50-99-7	Glucose	0.20	1.0E-03
57-48-7	Fructose	0.22	5.0E-02
603-06-3	Xylose	0.24	5.0E-02
9063-38-1	glycolate	0.40	5.0E-02
473-81-4	Glycerate (glyceric acid)	0.40	5.0E-02
6937-16-2	aminobutyrate	0.55	5.0E-02

*statistical changes were calculated automatically in BioSynLab software using pair-wise student t-tests and a minimum of three biological replicates (Enfissi *et al.,* 2010)

Supplementary Table S2, *List of significantly changed proteins in transgenic maize line Ph3*

**Accession**	**Description**	**Ratio Ph3 / WT**	**P-value**
tr|C0P9W6|C0P9W6_MAIZE	Uncharacterized protein	16.66	2.06E-02
GRMZM2G012071_P01	Uncharacterized protein	6.25	9.61E-03
tr|C0PC62|C0PC62_MAIZE	Uncharacterized protein	4.55	2,42E-05
tr|C4J4E4|C4J4E4_MAIZE	Uncharacterized protein	3.57	1.38E-02
GRMZM2G027875_P01	Uncharacterized protein	2.94	8.19E-03
tr|B4FWP0|B4FWP0_MAIZE	Fructose-bisphosphate aldolase	2.54	3.43E-02
tr|K7V5E8|K7V5E8_MAIZE	Uncharacterized protein	2.38	9.49E-03
tr|K7VWJ6|K7VWJ6_MAIZE	Uncharacterized protein	2.22	1.76E-03
tr|Q9SAZ6|Q9SAZ6_MAIZE	Phosphoenolpyruvate carboxylase	2.12	1.65E-03
tr|Q5EUD5|Q5EUD5_MAIZE	Protein disulfide isomerase	2.13	1.29E-02
GRMZM5G852504_P01	Uncharacterized protein	0.17	4.32E-02
tr|B8YDC0|B8YDC0_MAIZE	Pyruvate orthophosphate dikinase	0.23	2.17E-04
tr|K7VVI6|K7VVI6_MAIZE	Pyruvate, phosphate dikinase	0.25	2.01E-03
sp|P01073|ITRY_MAIZE	Trypsin inhibitor	0.26	4.72E-02
tr|C0LNQ9|C0LNQ9_MAIZE	UDP-glucosyltransferase	0.27	8.33E-03
tr|B4F9G8|B4F9G8_MAIZE	Pyruvate kinase	0.34	2.62E-02
tr|K7UPU2|K7UPU2_MAIZE	Uncharacterized protein	0.38	2.90E-02
tr|K7UMT9|K7UMT9_MAIZE	Uncharacterized protein	0.39	1.15E-03
tr|B6TIK6|B6TIK6_MAIZE	Sarcosine oxidase	0.39	2.52E-02
tr|C0P790|C0P790_MAIZE	Eukaryotic translation initiation factor 3	0.42	7.42E-04
GRMZM2G157061_P01	Uncharacterized protein	0.42	3.48E-03
AC212086.4_FGP003	Uncharacterized protein	0.46	5.34E-03
GRMZM2G124353_P01	Uncharacterized protein	0.46	1.42E-02
tr|K7UVD7|K7UVD7_MAIZE	Uncharacterized protein	0.48	6.40E-03

Supplementary Table S3, *List of 10 highest up and down changing transcripts in transgenic maize line Ph3*

**Accession**	**Description**	**Ratio Ph3 / WT**	**P-value**
GRMZM2G051943_P01	ATEP3| chitinase	47.53	4.45E-03
GRMZM2G125032_P01	hydrolase hydrolyzing O-glycosyl compounds	45.36	4.28E-04
Zm.14036.1.S1_at*	Uncharacterized protein	18.78	6.51E-03
GRMZM2G162659_P01	embryo specific protein5	16.04	5.01E-03
GRMZM2G374971_P01	hypothetical LOC542299	15.51	6.57E-03
Zm.4270.2.A1_a_at*	Uncharacterized protein	14.57	2.81E-03
Zm.105.1.S1_at*	Uncharacterized protein	14,04	2.91E-03
Zm.8822.2.A1_at*	Uncharacterized protein	13,95	7.55E-04
GRMZM2G374971_P01	ATOSM34 (osmotin 34)	13.65	5.47E-03
Zm.14496.1.A1_at*	(S)-beta-macrocarpene synthase	13.28	4.51E-02
GRMZM2G447984_P01	histone H3	0.17	1.06E-04
GRMZM2G026346_P02	cyclin-dependent protein kinase regulator	0.20	3.22E-05
GRMZM2G475899_P01	histone H3	0.21	2.71E-03
GRMZM2G179005_P03	histone H3	0.23	2.96E-04
GRMZM2G028955_P01	HTA6| DNA binding	0.24	6.15E-05
GRMZM2G323679_P01	oxidoreductase/ ribonucleoside-diphosphate reductase	0.25	2.59E-05
GRMZM2G479684_P01	histone H4	0.26	2.22E-04
GRMZM2G109448_P01	HTA12| DNA binding	0.28	1.54E-03
GRMZM2G125648_P01	HMG type nucleosome/chromatin assembly factor D	0.29	3.05E-04
GRMZM2G389958_P01	histone H3	0.31	1.21E-04

*Affymetrix ID
